# Periodontal Healing after Lower Third Molars Extraction: A Clinical Evaluation of Different Flap Designs

**DOI:** 10.3390/healthcare10081587

**Published:** 2022-08-21

**Authors:** Valentina Castagna, Alessia Pardo, Luca Lanaro, Annarita Signoriello, Massimo Albanese

**Affiliations:** 1 Dentistry and Maxillofacial Surgery Unit, Department of Surgery, Dentistry, Paediatrics and Gynaecology (DIPSCOMI), University of Verona, Piazzale L.A. Scuro 10, 37134 Verona, Italy; 2 Unit of Maxillofacial Surgery, Dell’Angelo Hospital, Via Paccagnella 11, 30174 Venezia, Italy

**Keywords:** extraction, periodontal, third molar

## Abstract

Periodontal healing after third molars extraction seems to be influenced by the choice of different flap techniques. The purpose of the present study was to assess the clinical condition of adjacent first and second molar sites, after the extraction of lower third molars, performed through different flap designs. Eighty patients, aged between 14 and 30 years, were analyzed for periodontal parameters of VPI, PPD, and CAL, pre-operatively (T0), after 15 days (T1), after 1 month (T2), and after 2 months (T3) from extraction. Techniques performed were trapezoidal flap (TRAP), marginal flap (MARG), flap with papilla detachment (DETP), and flap with papilla decapitation (DEC). No significant differences were found between the four flaps at each observation time and considering the interval between T0 and T3, for VPI, PPD at first molar site, PPD at second molar site, and CAL at second molar site. Significant variations for CAL were registered, for each flap, between T0 and T3, in all cases for buccal site, in three cases for buccal-distal site. After 2 months of follow-up, no strong evidence can be assumed for or against the use of a particular flap design for the extraction of lower third molars.

## 1. Introduction

The most frequent indication for the extraction of third molars is dysodontiasis [1], which concerns alterations related to inclusion, to an eruptive deficit during dental roots development, and to the lack of dragging competence by periodontal ligament [2]. In this regard, assessing the level of inclusion of third molars in the bone and in the soft tissues is fundamental for a complete pre-operative examination [3].

As important as the pre-clinical evaluation, the post-surgical phase raises relevant issues [4], directly connected to the maintenance of periodontal health of post-surgical sites of the adjacent first and second molars, often involved in surgical incisions design: an accurate evaluation of these sites allows to assess possible gingival defects or periodontal pockets. Several studies in literature [4,5,6,7,8] frequently reported the following complications after third molars surgical extraction: presence of periodontal pockets, bone loss, or loss of clinical attachment level (CAL), especially at the buccal/distal sides of second molar site. Differently from transient outcomes as post-operative pain sensations and swelling, the development of severe periodontal defects in post-surgical sites are often characterized by a chronic course, linked to plaque accumulation, stable local inflammatory signs, and bone resorption [9].

In this proposal, it seems that the choice of a specific surgical flap design represents a significant factor interfering with post-surgical periodontal healing [6]. Among several types of flap design proposed for the extraction of lower third molars, the most frequently cited are envelope, triangular, trapezoidal, and paramarginal [10,11,12]. Regarding their influence on soft tissues healing, an important review [3] has shown that no specific technique significantly leads to remarkable reductions of post-operative probing pocket depth (PPD) or CAL. Nevertheless, some flap designs (i.e., Szmyd technique and paramarginal flap) seem to be more effective in causing irrelevant variations of PPD after the extraction of included third molars, while others (i.e., envelope flap) seem to demonstrate worse outcomes in terms of periodontal healing [3].

Given these considerations, the aim of this study was to investigate potential differences in terms of periodontal healing, at first and second lower molar sites, after surgical extraction of lower third molars. A muco-periosteal flap was always performed in all techniques to assure the surgical goal, conserve pre-operative anatomical conditions, and minimize at the same time both periodontal and aesthetic post-operative complications.

## 2. Materials and Methods

### 2.1. Study Sample

A study was conducted as a retrospective evaluation, between January 2019 and February 2020, on the available medical records, possible to evaluate for satisfaction of the following inclusion criteria and with a 2-months follow-up after extraction, of 80 patients, aged between 14–30 years [13] and previously involved (during 2018) in lower third molar surgery at the Dentistry and Maxillo-facial Surgery Unit (University of Verona). Subjects included in the study presented ASA (American Society of Anesthesiology classification) status I, that is negative history for systemic diseases and known drug therapies; possible organic, physiological, biochemical, or psychiatric disorders were thus excluded from the study. Smoking habits, previous or current chemotherapy and radiotherapy, and patients who had a post-extraction abscess after surgery or post-operative alveolitis were also considered as exclusion criteria.

The nature and aim of the study, together with the anonymity in the scientific use of data, were clearly presented in a written informed consent form, obtained from all subjects involved in the study; appropriate forms were regularly filled in by parents or related legal guardians also for patients <18 years. The study was also conducted in accordance with the Declaration of Helsinki and good clinical practice guidelines for research on human beings. The University Institutional Review Board approved the retrospective study (Protocol “POST-ESTR.LEMBI”, Prog. 3921CESC). The study presents compliance with the STROBE checklist guidelines [14].

### 2.2. Surgical Techniques

All surgical procedures were carried out by the same surgeon. A complete pre-operative evaluation was conducted for degree of inclusion of lower third molar (included, semi-included, or extruded); relationship with the second molar and with the antagonist upper molar; absence or presence of inflammation and carious lesions, both evaluated for third molar and second molar. Pell and Gregory classification was used to classify the positions of third molars (Class I, II, III according to the relationship to the anterior border of mandibular ramus; Class A, B, C according to the relationship to the occlusal plane of the adjacent second molar) [15]. Cone-beam computed tomography (CBTC) was evaluated for each patient [16]. In case of bilateral cases of mandibular third molars in the same patient, they were both removed during one-time surgery.

One gram of Amoxicillin + Clavulanic Acid was orally administered 24 h before surgery, and Chlorhexidine mouthwash 0.12% was administered for one minute one hour before surgery. Local anesthesia was used as troncular nerve block (Articaine 1:100,000 without Adrenaline) and plexus block (Articaine 1:100,000 with Adrenaline).

Soft tissue incisions had to guarantee a visible section of epithelial, connective tissue and periosteal layers to assure proper wound closure in flaps repositing and to allow first wound healing; furthermore, excessive surgical trauma and lacerations of the flap edges were avoided. Scalpel blade N.15 (Hu-Friedy Italy Srl, Milano, Italy) was used for soft tissues incisions and consequent sub-periosteal dissection.

Flaps techniques used are described as follows. All flap designs presented a distal releasing incision, with a distal-buccal direction, that is an angulation of 45 degrees to the ideal prosecution of dental arch [17]. Furthermore, all flap incisions involved both first and second molars.

- Technique I—Trapezoidal flap (TRAP) [18]

**Figure 1:**
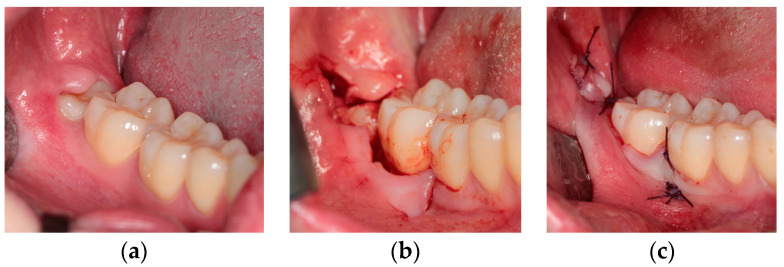
Trapezoidal flap: (**a**) pre-operative clinical view; (**b**) intra-operative incisions; (**c**) final suture.

This flap (Figure 1) provides an intrasulcular-buccal incision at the level of adherent gingiva, which starts from the central-buccal side of first molar clinical crown, then continues at the level of second molar, and finally ends with a releasing incision towards the distal-buccal side of second molar. This flap associates another mesial releasing incision (starting from the distal-buccal gingival margin of first molar), which allows an easier flap dissection compared to the other techniques.

- Technique II—Marginal flap (envelope) (MARG) [3]

**Figure 2:**
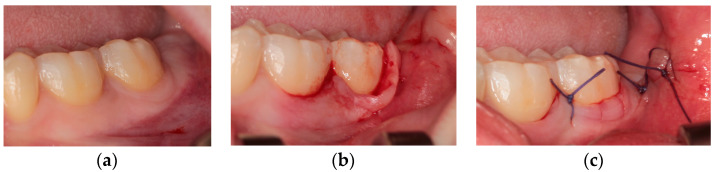
Marginal flap: (**a**) pre-operative clinical view; (**b**) intra-operative incisions; (**c**) final suture.

This flap (Figure 2) provides the same incision as the previous one, but no mesial incisions are proposed in this case. For this reason, the final sub-periosteal flap dissection is difficult to perform, especially in presence of a thin periodontal phenotype.

- Technique III—Flap with papilla detachment (DETP)

**Figure 3:**
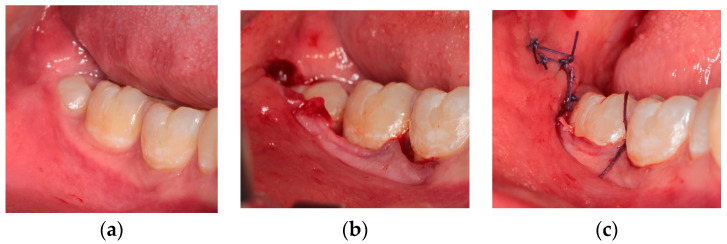
Flap with papilla detachment: (**a**) pre-operative clinical view; (**b**) intra-operative incisions; (**c**) final suture.

This flap (Figure 3) can be considered a modification of the envelope design [3]: it is characterized for the use of the periosteal elevator, followed by the scalpel. Incision starts by inserting the periosteal elevator inside the gingival sulcus along the central-buccal side of first molar clinical crown, then continues distally, detaching the gingival papilla between first and second molar, with detachment of periodontal ligament also from the second molar. Only the final releasing incision is performed with the scalpel, starting distally from the distal side of second molar, with a distal-buccal direction.

- Technique IV—Flap with papilla decapitation (DEC)

**Figure 4:**
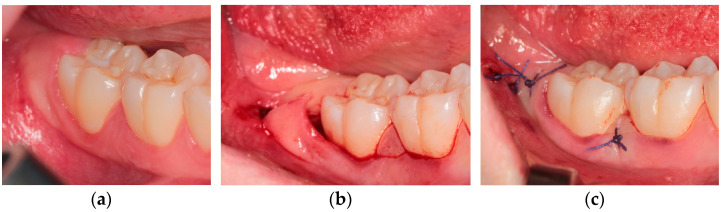
Flap with papilla decapitation: (**a**) pre-operative clinical view; (**b**) intra-operative incisions; (**c**) final suture.

This flap (Figure 4) can be also considered a modification of the envelope design [3]. Intrasulcular incision starts from the first molar, continues parallel to the occlusal plane in the gingival papilla between first and second molar, and through the gingival sulcus of second molar, finally ending with the distal-buccal releasing incision in common with the other flaps described above [17].

### 2.3. Soft Tissues Assessment

Periodontal soft tissues were assessed using a periodontal probe (Florida Probe; Florida Probes Company, Gainesville, FL, USA), applying a force of mild intensity. Parameters considered were [19,20]:

Probing Pocket Depth (PPD), recorded in mm as the distance between the gingival margin and the base of periodontal pocket;

Clinical Attachment Loss (CAL), recorded in mm as the distance from the cementum-enamel junction (CEJ) to the location of probe tip;

Visible Plaque Index (VPI), recorded as 0 (no plaque) or 1 (plaque) after probing for PPD.

Clinical soft tissues examination [3,4,6,12] was conducted for first molar and second molar sites, focusing on the degree of healing of surgical flaps in the most critical sites. At this proposal, the following sites were finally registered:

- First molar buccal site PPD, calculated in mm as an average value of mesial, central, and distal sites explored on buccal side of first molar;
- Second molar buccal site PPD/CAL, calculated in mm as an average value of mesial, central, and distal sites explored on buccal side of second molar;
- Second molar distal site VPI, expressed in % as an average value recorded on buccal-distal, central-distal, and lingual-distal sites explored on distal side of second molar;
- Second molar distal site PPD, recorded in mm as an average value recorded on buccal-distal, central-distal, and lingual-distal sites explored on distal side of second molar;
- Second molar buccal-distal site CAL, recorded in mm.

All sites were detected four times: pre-operatively (T0), after 15 days (T1), after 1 month (T2), and after 2 months (T3).

Moreover, an overall clinical evaluation was conducted at T0 to register general periodontal conditions [21].

### 2.4. Statistical Analysis

For data collection, a database including all patients evaluated in the study was created with Microsoft Excel. All data analysis was carried out using Stata v.13.0 for Macintosh (StataCorp, College Station, TX, USA). The normality assumptions for continuous data were assessed by using the Shapiro–Wilk test; mean and standard deviation were reported for normally distributed data, median, and interquartile range (iqr) otherwise. For categorical data, absolute frequencies, percentages, and 95% confidence intervals were reported. The comparison between the means of continuous variables in different times was performed by using paired Student’s *t* test or Wilcoxon matched-pairs signed-rank test. The comparison of the means among groups was done using one-way analysis of variance (ANOVA), or Kruskal–Wallis equality-of-populations rank test. Significance level was set at 0.05.

## 3. Results

### 3.1. Study Sample

A total of 80 (31 men and 49 women) out of 88 eligible patients were finally included in the study: 8 patients who presented post-operative alveolitis did not meet the inclusion criteria (one case also required a surgical revision of the alveolar socket). Mean age was 22 (14–30 years). Teeth were surgically extracted with the following techniques respectively: TRAP in 18 patients, MARG in 28 patients, DETP in 20 patients, and DEC in 14 patients. As the study was conducted between January 2019 and February 2020, it was not possible to evaluate homogeneous groups of flap designs, due to the COVID-19 pandemic’s impact on clinical activities.

Demographics is reported in Table 1.

Regarding the overall periodontal conditions (Table 1), none of the patients presented any forms of periodontitis, while 13 showed gingivitis. Considering the overall sample, PPD values of ∆ (T0–T3) at second molar sites resulted to be significantly different among healthy patients and patients with gingivitis; furthermore, comparing flaps techniques, DEC and MARG showed significant differences for PPD and VPI values of ∆ (T0–T3) at second molar sites (Table 2).

Concerning tooth class described as degree of inclusion (Table 1), no significant differences, both for overall sample and comparing flaps techniques, were assessed for PPD, VPI, or CAL values of ∆ (T0–T3) at first or second molar sites (Table 3).

### 3.2. Soft Tissues Assessment

To focus on the most important clinical variations after 2 months, PPD was described at T0, T1, T2, and T3, while VPI and CAL were described at T0 and T3.

#### 3.2.1. Visible Plaque Index

Table 4 reports outcomes of mean VPI, assessed for second molar distal site: a mean slight increase of 0.01 was registered between T0 and T3. No significant differences between the four flaps were found at each observation time and considering the interval between T0 and T3.

#### 3.2.2. Probing Pocket Depth at First Molar Site

Mean values assessed for first molar buccal site (Table 5) demonstrated a moderate increase between T0 and T1, followed by a gradual slight decrease at T2 and finally at T3. No statistical differences were found between the four flaps at each observation time and considering the interval between T0 and T3. However, the overall variation between T0 and T3 was registered as statistically significant (*p* = 0.002), for a value of 0.13 mm (from 1.65 mm to 1.79 mm); similarly, technique MARG registered a statistically significant variation (*p* = 0.04) between T0 and T3, for a mean value of 0.16 mm. From a clinical point of view, these significant values cannot be considered as particularly relevant in terms of PPD, as both variations are very small.

#### 3.2.3. Probing Pocket Depth at Second Molar Site

Mean values assessed for second molar buccal site (Table 6) demonstrated an evident increase between T0 and T1, followed by a gradual decrease at T2 and finally at T3. Statistical differences were found between the four flaps only at T0 (*p* = 0.003). Variation between T0 and T3 was registered as statistically significant (*p* = 0.004), for an overall value of 0.19 mm (from 2.34 mm to 2.53 mm); similarly, technique DETP registered a statistically significant variation (*p* = 0.04) between T0 and T3, for a mean value of 0.31 mm. From a clinical point of view, these values can be considered as moderately relevant in terms of PPD.

Mean values assessed for second molar distal site (Table 7) demonstrated a large increase between T0 and T1, followed by a gradual decrease at T2 and finally at T3. No statistical differences were found between the four flaps at each observation time and considering the interval between T0 and T3. Despite that, technique MARG showed a smaller variation (0.05 mm) between T0 and T3, compared to the other flaps. Variation between T0 and T3 was registered as statistically significant (*p* = 0.01), for an overall value of 0.45 mm (from 2.89 mm to 3.34 mm), which, from a clinical point of view, can be considered as relevant in terms of PPD.

#### 3.2.4. Clinical Attachment Loss at Second Molar Site

Table 8 and Table 9 report outcomes for CAL, assessed for second molar site respectively at buccal and buccal-distal surfaces. In both cases, overall statistically significant variations (*p* = 0.0001) were registered between T0 and T3 (0.63 mm and 0.84 mm for buccal and buccal-distal surface respectively). No significant differences were found between the four flaps at each observation time and considering the interval between T0 and T3. However, significant variations were registered for each flap considering the interval between T0 and T3, in all cases for buccal site, in three cases for buccal-distal site: from a clinical point of view, these values can be all considered as relevant in terms of CAL.

## 4. Discussion

Several authors [4,6,8] reported that the choice of specific surgical flap designs used to perform the extraction of lower third molars can influence the visibility/accessibility of the surgical site and interfere with post-operative healing. These surgical interventions should be conducted with strong attempt, based on the best evidence combined with clinical experience, to reduce biological costs for the patient, especially if they are asymptomatic. Some techniques can be thus addressed to minimize post-operative complications: for example, envelope design seems to be effective in reducing post-operative discomfort, trismus, pain and swelling [18].

On the other hand, some studies in literature [4,6,12,22,23,24] declared no relevant influence of specific flap designs on periodontal health of the adjacent second molar after the extraction of impacted third molar. Nevertheless, it is difficult to generalize these considerations because of different short and long-term follow-ups assessed. Furthermore, an issue which represents a strong limit for comparing homogeneously available outcomes is that the main periodontal parameters were not always measured according to the same methodology. Rosa et al. [6], comparing 3-cornered flap and Szmyd flap, showed that both flaps caused, with no significant differences, periodontal complications to the adjacent second molar, with an increase in probing depth, clinical attachment loss, and bone level resorption, found at 3 and 6 months post-operatively. Kirtiloğlu et al. [23] demonstrated significantly different mean probing depths at distal and buccal sites of second molar site, for two flap designs at 1-week, 2-week, and 4-week intervals; no significant differences between pre-operatively and 1-year follow-up were otherwise found for mean values of PD and CAL. Arta et al. [24], comparing two flaps (Szmyd and triangular flaps), revealed similar results achieved after 2 weeks, 4 weeks, and 6 months.

The distal site of second molar is considered a critical surface, at greater risk for the development of notable iatrogenic post-surgical periodontal pockets, with great increase of PPD and CAL values [25]. In addition, first molar should also be evaluated for its usual involvement in the incisions [3]. Overall values found for PPD in our study could be considered acceptable from a clinical point of view and comparable with other studies in literature with a similar follow-up [4]. These values increase 15 days after extraction and then gradual decrease, until a final stabilization, tending to overlap to the initial pre-surgical phase. The biggest variation for PPD was found for second molar distal site, which presented an overall statistically significant variation of 0.45 mm between T0 and T3: this value appears relevant in terms of PPD and clinically compatible with physiological healing times 2 months after extraction. Technique MARG particularly showed a smaller variation (0.05 mm) between T0 and T3, compared to the other flap designs. Similar PPD variations were reported by other authors [22], confirming a general post-operative PDD increase, ranging from 0.02 to 3.33 mm, within 2 weeks of follow-up, even though different flap designs were compared. Arta et al. [24] presented values of PPD of 1.65 mm at baseline and at 2 weeks for 3-cornered flap, 1.73 mm at baseline and 1.75 mm at 2 weeks for Szmyd flap. Monaco et al. [26] proposed triangular and envelope flaps, showing an increase in probing depths for all teeth examined 7 days after extraction; greater significant values were recorded for PPD of first and second molars in case of envelope flap compared to the triangular one. Stephens et al. [22] analyzed envelope and modified envelope flaps, finding no significant differences between them pre-operatively and after 12 weeks; an improvement of probing depth on three surfaces of second molars after 3 months was also reported. Quee et al. [27] showed greater values on the distal surfaces at 6 months post-operatively.

Concerning CAL, significant variations were registered in our study for each flap considering the interval between T0 and T3, in all cases for buccal site, in three cases for buccal-distal site: from a clinical point of view, these values can be considered relevant in terms of CAL, as a remarkable increase is usually expected 2 months after surgery. As confirmed by other investigations [6,23], no significant CAL values were found in comparing different flap techniques.

Indexes of gingival inflammation, i.e., plaque index, although considered as risk factors for a delayed healing of post-surgical wound, do not remarkably affect overall periodontal conditions [4]. This is also confirmed by our results: VPI values assessed for second molar distal site showed no significant differences between the four flaps at each observation time and considering the interval between T0 and T3.

To sum up, from data obtained in the present study, apart from statistical considerations, the flap designs which seemed advantageous for periodontal healing from a clinical point of view (lowest variations between T0 and T3), were:

- DEC and DETP flap, in terms of PPD of first molar site (buccal);
- MARG flap, in terms of PPD of second molar sites (buccal and distal).

If we look at CAL variations for second molar site, the best performances with the lowest values between T0 and T3 (considering respectively variations of buccal and buccal-distal sites) was demonstrated by DETP and TRAP flap.

Concerning the worst performances, they were shown by:

- TRAP flap, in terms of PPD of first molar site (buccal);
- DEC flap, in terms of PPD of second molar sites (buccal and distal);
- MARG flap, in terms of CAL of second molar sites (buccal and buccal-distal).

Some authors [18] claimed that the greatest loss of periodontal attachment is usually shown by trapezoidal flap: this is probably due to the peculiarity of this flap design, which lies in the mesial discharge incision. In terms of periodontal healing, a greater laxity of the wound is unfortunately present, with the consequence of greater loss of attachment compared to the other flaps. This issue is indeed confirmed by our results regarding PPD values for first molar site. On the other side, the repositioning of this flap can be easily obtained since the releasing incision at the level of the central-buccal part of the first molar preserves the papilla and facilitates the suture.

Considering the other flaps proposed in the present study, it can be said that outcomes did not demonstrate big differences between techniques used in terms of positive or negative performances, which were respectively found for each flap in terms of PPD or CAL. Furthermore, despite the design, some flaps differed from each other for instruments of execution: DETP flaps showed good performances in terms of PPD and CAL, with a minimum clinical increase respectively at the level of first and second molar. It would be reasonable to assume that other peri-operative factors, such as suture or ostectomy methods, might also influence periodontal health: in the DEC technique, a greater consideration for the interdental papilla between first and second molar is evident; in terms of suture, since the mobile flap approaches the papilla as a fixed point, a better healing of the periodontium after surgical extraction can be thus observed [28].

Regarding the influence of overall pre-operative clinical conditions on periodontal healing, significant differences were found for PPD and VPI values of ∆ (T0–T3) at second molar sites, both for overall sample and for comparison between techniques: this consideration led the authors to assume these outcomes as minimally relevant from a clinical point of view, considering that none of the patients presented any forms of periodontitis, with only 13 cases of gingivitis, and significant variations did not concern CAL.

Analysis of soft tissues indexes of periodontal healing between T0 and T3, according to different pre-operative degree of inclusion, for overall sample and comparing different flaps techniques, did not reveal any significant differences between groups. Despite the non-homogeneous distribution of tooth class, our results seem to suggest that degree of inclusion does not represent a critical issue on periodontal healing after surgery apart from the technique used. On the other hand, other authors [8] reported a significant impact of position B and C on periodontal defects if different techniques for tooth removal are used, with better healing for distolingual alveolectomy using a chisel instead of tooth division with burs. Nevertheless, comparison between studies does not offer a complete interpretation of outcomes, as surgical procedures proposed are various, plus analysis according to degree of inclusion as a main aspect is not always performed [6].

An extent of follow-up may be advisable for proper comparison with other studies, as our two-month follow-up represents a limitation, together with the non-homogeneous distribution of flap design groups considered, due to the COVID-19 pandemic’s impact on clinical activities. Finally, the presence of reparative tissue at the most distal site of second molar refers to a wound healing which does not always completely restore the architecture of this site: the repair function can be characterized by a long junctional epithelium, possible to evaluate only through histological analysis [29]. However, a possible strength for this study is pointed out by the detailed assessment of soft tissues indexes, on multiple surfaces of both first and second molar adjacent sites.

## 5. Conclusions

Outcomes of the present study do not provide convincing evidence for the controversy over the use of a specific flap design for surgical extraction of lower third molars. Considering the trend of PPD and CAL obtained for adjacent first and second molar sites, techniques analyzed did not demonstrate significant differences between each other in terms of influence on periodontal healing. It should be noted, for all flaps, an overall increase in PPD during the first two post-operative weeks, with a return to pre-operative values within the following 2 months. However, significant variations for CAL were registered, for each flap, between T0 and T3, in all cases for buccal site, in three cases for buccal-distal site of second molar.

In light of these considerations, authors suggest preventing and minimizing biological risks associated with the extraction of third molars by performing a surgery which is highly respectful towards periodontal tissues.

## Figures and Tables

**Table 1:** Patients and tooth characteristics at baseline.

Variable	*n*	%
Sex		
male	31	38.75
female	49	61.25
Age ^1^	22 ± 4.22	years
Tooth element		
element 38	46	57.5
element 48	34	42.5
Tooth class (Pell and Gregory classification)		
IA	15	18.75
IIA	10	12.5
IB	18	22.5
IIB	19	23.75
IIIB	5	6.25
IC	1	1.25
IIC	6	7.5
IIIC	6	7.5
Overall periodontal conditions		
gingival health	67	83.75
moderate gingivitis	10	12.5
severe gingivitis	3	3.75

^1^ Age is presented as mean (±standard deviation).

**Table 2:** Analysis of soft tissues indexes of periodontal healing between T0 and T3, according to pre-operative periodontal diagnosis, for overall sample and comparing different flaps techniques; *p* values are reported.

OVERALL PERIODONTAL DIAGNOSIS (Healthy, Moderate Gingivitis, Severe Gingivitis)			FIRST MOLAR BUCCAL SITE PPD ∆ (T0–T3)	SECOND MOLAR BUCCAL SITE PPD ∆ (T0–T3)	SECOND MOLAR DISTAL SITE PPD ∆ (T0–T3)	SECOND MOLAR DISTAL SITE PLAQUE INDEX ∆ (T0–T3)	SECOND MOLAR BUCCAL SITE CAL ∆ (T0–T3)	SECOND MOLAR BUCCAL-DISTAL SITE CAL ∆ (T0–T3)
			*p* value	*p* value	*p* value	*p* value	*p* value	*p* value
		OVERALL	0.74	0.001 *	0.001 *	0.34	0.53	0.42
	TECHNIQUE							
		*DEC*	0.88	0.01 *	0.001 *	0.03 *	0.51	0.29
		*DETP*	0.61	0.25	0.13	0.54	0.94	0.48
		*MARG*	0.08	0.02 *	0.04 *	0.01 *	0.13	0.85
		*TRAP*	0.28	0.22	0.20	0.86	0.17	0.19

*: statistically significant differences between groups.

**Table 3:** Analysis of soft tissues indexes of periodontal healing between T0 and T3, according to pre-operative degree of inclusion, for overall sample and comparing different flaps techniques; *p* values are reported.

DEGREE OF INCLUSION (IA, IIA, IB, IIB, IIIB, IC, IIC, IIIC)			FIRST MOLAR BUCCAL SITE PPD ∆ (T0–T3)	SECOND MOLAR BUCCAL SITE PPD ∆ (T0–T3)	SECOND MOLAR DISTAL SITE PPD ∆ (T0–T3)	SECOND MOLAR DISTAL SITE PLAQUE INDEX ∆ (T0–T3)	SECOND MOLAR BUCCAL SITE CAL ∆ (T0–T3)	SECOND MOLAR BUCCAL-DISTAL SITE CAL ∆ (T0–T3)
			*p* value	*p* value	*p* value	*p* value	*p* value	*p* value
		OVERALL	0.75	0.46	0.26	0.88	0.52	0.54
	TECHNIQUE							
		*DEC*	0.42	0.52	0.25	0.68	0.65	0.44
		*DISPAP*	0.49	0.80	0.40	0.05	0.30	0.20
		*MARG*	0.66	0.33	0.88	0.36	0.99	0.74
		*TRAP*	0.73	0.26	0.15	0.36	0.17	0.59

**Table 4:** Second molar distal site plaque index. Values are presented in %.

		T0	T3	∆ (T0–T3)	*p* Value
	OVERALL	0.13 (0.34)	0.14 (0.35)	0.01 (0.43)	0.79
TECHNIQUE					
	*DEC*	0.06 (0.25)	0.18 (0.40)	0.12 (0.50)	0.31
	*DETP*	0.15 (0.36)	0.25 (0.44)	0.10 (0.30)	0.15
	*MARG*	0.11 (0.32)	0.11 (0.32)	0.01 (0.39)	0.99
	*TRAP*	0.22 (0.42)	0.05 (0.23)	0.16 (0.51)	0.17
	*p* value	0.56	0.34	0.17	

**Table 5:** First molar buccal site PPD. Values are presented in mm as mean (± standard deviation).

		T0	T1	T2	T3	∆ (T0–T3)	*p* Value
	OVERALL	1.65 ± 0.45	1.98 ± 0.48	1.84 ± 0.44	1.79 ± 0.42	0.13 ± 0.43	0.002 *
TECHNIQUE							
	*DEC*	1.79 ± 0.44	2.03 ± 0.49	1.94 ± 0.45	1.83 ± 0.38	0.09 ± 0.37	0.06
	*DETP*	1.53 ± 0.48	1.94 ± 0.54	1.82 ± 0.38	1.62 ± 0.41	0.09 ± 0.45	0.59
	*MARG*	1.61 ± 0.37	1.98 ± 0.45	1.79 ± 0.39	1.77 ± 0.43	0.16 ± 0.44	0.04 *
	*TRAP*	1.76 ± 0.52	1.98 ± 0.50	1.85 ± 0.54	1.96 ± 0.41	0.19 ± 0.46	0.06
	*p* value	0.42	0.82	0.63	0.07	0.71	

* statistically significant differences between groups/observation times.

**Table 6:** Second molar buccal site PPD. Values are presented in mm as mean (± standard deviation).

		T0	T1	T2	T3	∆ (T0–T3)	*p* Value
	OVERALL	2.34 ± 0.65	3.79 ± 1.06	2.94 ± 0.70	2.53 ± 0.62	0.19 ± 0.64	0.004 *
TECHNIQUE							
	*DEC*	2.18 ± 0.65	3.72 ± 1.27	3.00 ± 0.66	2.57 ± 0.76	0.38 ± 0.75	0.11
	*DETP*	1.96 ± 0.50	3.51 ± 0.86	2.72 ± 0.68	2.28 ± 0.66	0.31 ± 0.77	0.04 *
	*MARG*	2.49 ± 0.62	3.98 ± 1.15	2.87 ± 0.76	2.56 ± 0.43	0.03 ± 0.53	0.19
	*TRAP*	2.67 ± 0.65	3.86 ± 0.94	3.22 ± 0.62	2.74 ± 0.66	0.06 ± 0.47	0.46
	*p* value	0.003 *	0.54	0.12	0.14	0.80	

* statistically significant differences between groups/observation times.

**Table 7:** Second molar distal site PPD. Values are presented in mm as mean (± standard deviation).

		T0	T1	T2	T3	∆ (T0–T3)	*p* Value
	OVERALL	2.89 ± 1.37	5.64 ± 2.49	4.33 ± 1.68	3.34 ± 1.35	0.45 ± 1.43	0.01 *
TECHNIQUE							
	*DEC*	2.50 ± 1.41	5.75 ± 3.31	4.68 ± 2.18	3.37 ± 1.62	0.87 ± 1.74	0.16
	*DETP*	2.35 ± 0.74	4.92 ± 1.47	3.75 ± 1.40	2.95 ± 1.43	0.60 ± 1.50	0.19
	*MARG*	3.31 ± 1.46	6.03 ± 2.27	4.29 ± 1.43	3.37 ± 1.11	0.05 ± 1.31	0.55
	*TRAP*	3.22 ± 1.51	5.77 ± 2.88	4.72 ± 1.74	3.72 ± 1.31	0.50 ± 1.20	0.08
	*p* value	0.06	0.56	0.23	0.19	0.68	

* statistically significant differences between groups/observation times.

**Table 8:** Second molar buccal site CAL. Values are presented in mm as mean (± standard deviation).

		T0	T3	∆ (T0–T3)	*p* Value
	OVERALL	2.70 ± 1.09	3.33 ± 0.99	0.63 ± 0.87	0.0001 *
TECHNIQUE					
	*DEC*	2.71 ± 1.09	3.43 ± 0.83	0.71 ± 0.94	0.005 *
	*DETP*	2.77 ± 1.04	3.15 ± 0.89	0.37 ± 0.66	0.01 *
	*MARG*	2.42 ± 1.08	3.16 ± 1.22	0.74 ± 0.92	0.001 *
	*TRAP*	3.02 ± 1.14	3.72 ± 0.75	0.69 ± 0.95	0.01 *
	*p* value	0.37	0.17	0.39	

* statistically significant differences between groups/observation times.

**Table 9:** Second molar buccal-distal site CAL. Values are presented in mm as mean (± standard deviation).

		T0	T3	∆ (T0–T3)	*p* Value
	OVERALL	3.91 ± 1.20	4.76 ± 1.85	0.84 ± 1.61	0.0001 *
TECHNIQUE					
	*DEC*	3.93 ± 1.28	4.56 ± 2.27	0.62 ± 1.92	0.22
	*DETP*	3.75 ± 1.33	4.57 ± 1.53	0.82 ± 1.33	0.01 *
	*MARG*	3.90 ± 1.20	5.05 ± 2.10	1.14 ± 1.90	0.001 *
	*TRAP*	4.11 ± 1.02	4.72 ± 1.40	0.61 ± 1.09	0.04 *
	*p* value	0.80	0.68	0.46	

* statistically significant differences between groups/observation times.

## Data Availability

The data presented in this study are available on request from the corresponding author.
